# Advances in Genotyping Detection of Fragmented Nucleic Acids

**DOI:** 10.3390/bios14100465

**Published:** 2024-09-28

**Authors:** Qian Liu, Yun Chen, Hao Qi

**Affiliations:** 1 School of Chemical Engineering and Technology, Tianjin University, Tianjin 300350, China; liuqian5765@tju.edu.cn (Q.L.); chenyun823@tju.edu.cn (Y.C.); 2 Key Laboratory of Systems Bioengineering (Ministry of Education), School of Chemical Engineering and Technology, Tianjin University, Tianjin 300072, China

**Keywords:** fragmented nucleic acids, single nucleotide variants, nucleic acid detection, liquid biopsy

## Abstract

Single nucleotide variant (SNV) detection is pivotal in various fields, including disease diagnosis, viral screening, genetically modified organism (GMO) identification, and genotyping. However, detecting SNVs presents significant challenges due to the fragmentation of nucleic acids caused by cellular apoptosis, molecular shearing, and physical degradation processes such as heating. Fragmented nucleic acids often exhibit variable lengths and inconsistent breakpoints, complicating the accurate detection of SNVs. This article delves into the underlying causes of nucleic acid fragmentation and synthesizes the strengths and limitations of next-generation sequencing technology, high-resolution melting curves, molecular probes, and CRISPR-based approaches for SNV detection in fragmented nucleic acids. By providing a detailed comparative analysis, it seeks to offer valuable insights for researchers working to overcome the challenges of SNV detection in fragmented samples, ultimately advancing the accurate and efficient detection of single nucleotide variants across diverse applications.

## 1. Introduction

In modern medical, food, and biotechnology fields, the detection of single nucleotide variants (SNVs) plays a pivotal role across various applications, such as disease diagnosis [1], viral detection [2,3], genetically modified organisms (GMOs) identification [4], and genotyping [5]. The human genome harbors a myriad of SNVs that are intricately linked to various diseases [6]. For example, mutations in the receptor tyrosine kinase protein ERBB family member EGFR are closely associated with lung adenocarcinoma [7]. In Asian patients with lung adenocarcinoma, these mutations account for 40–50% of cases [8], while in White patients, they are present in 10–20% of cases [9]. The COVID-19 pandemic caused by SARS-CoV-2 has underscored the significance of viral genomic variations, which continue to influence the transmissibility, pathogenicity, and immune evasion capabilities of the virus [2,10]. Rapid surveillance and analysis of these SNVs are crucial for tracking the evolution of SARS-CoV-2 variants worldwide, aiding real-time response efforts and mitigating the impact of future outbreaks [11]. SNV detection is instrumental in identifying GMOs and detecting unexpected genetic variations, enabling the assessment of their potential risks and environmental impacts [12]. It plays a pivotal role in the regulation, quality control, and biosafety assessment of GMOs, providing crucial technical support to ensure their safety and traceability.

However, with the increasing application of liquid biopsy and other non-traditional sample types, nucleic acids in these samples are often fragmented, posing challenges for accurate detection of single nucleotide variants. Fragmented nucleic acids can originate from a diverse array of sources, including but not limited to blood [13], saliva [14], urine [15], and physical degradation processes such as freeze–thaw cycles, pipetting, and vortexing. These nucleic acid fragments vary not only in abundance but also in length, ranging from tens to hundreds of base pairs. Nucleic acid fragmentation arises from multiple factors. A primary mechanism is apoptosis, during which nucleic acids are systematically fragmented. Free DNA (cfDNA) is present in bodily fluids, with tumor-derived cfDNA commonly referred to as circulating tumor DNA (ctDNA) [16]. ctDNA has garnered significant attention due to its role as a tumor biomarker [17]. The detection of ctDNA in patient plasma or serum allows for early cancer detection, determination of tissue origin, prognosis assessment, monitoring of treatment responses, and evaluation of potential drug resistance [18]. Similarly, circulatory endogenous single-stranded non-coding small RNA molecules (microRNA) also hold potential as biomarkers for cancer screening. These microRNAs can be secreted into the circulation and remain stable, often displaying abnormal expression profiles under various physiological and pathological conditions. The detection of these aberrantly expressed nucleic acid fragments can aid in cancer diagnosis [19,20]. Consequently, the development of advanced methodologies for detecting single-base mutations in fragmented nucleic acids is of significant clinical importance [21].

Here, we provide an overview of genotyping research on fragmented nucleic acids. First, we give a comprehensive summary of the mechanisms of nucleic acid fragmentation, followed by a discussion of various methods for detecting single nucleotide variants (SNVs) in nucleic acids. Additionally, we present our insights into the advantages, limitations, and development potential of fragmented nucleic acid detection technologies.

## 2. Mechanisms of Fragmented Nucleic Acid Molecule Formation

Nucleic acids used for SNV detection are often fragmented, a process influenced by various degradation mechanisms within cellular and external environments [22]. These fragments can originate from intracellular degradation processes, physical disruptions, or thermal degradation, each contributing uniquely to the complexity of nucleic acid analysis.

Intracellularly, nucleic acids are susceptible to degradation by nucleases, which cleave RNA and DNA molecules into smaller fragments. Mechanical stresses during sample processing, such as homogenization, centrifugation, or pipetting, can break nucleic acids into smaller fragments. Exposure to elevated temperatures also can induce the denaturation and subsequent fragmentation of nucleic acids. The non-uniformity in the locations of nucleic acid breaks, combined with the variability in fragment lengths, presents considerable challenges for SNV detection.

### 2.1. Intracellular Degradation

Liquid biopsy has become a key approach in oncology and diagnostics, offering the advantages of being non-invasive, sensitive, and dynamic compared to traditional tissue biopsy. Central to liquid biopsy is the detection of cell-free DNA (cfDNA), initially discovered by Mandel et al. in 1948 in the plasma of healthy and diseased individuals [23]. cfDNA, released into circulation primarily through cell necrosis or programmed cell death, includes circulating tumor DNA (ctDNA), which serves as a biomarker in liquid biopsy [24,25]. The length of cfDNA fragments varies depending on the tissue of origin; for instance, cfDNA from hematopoietic cells averages 166 bp, while cfDNA from placental cells measures around 143 bp.

**Figure 1:**
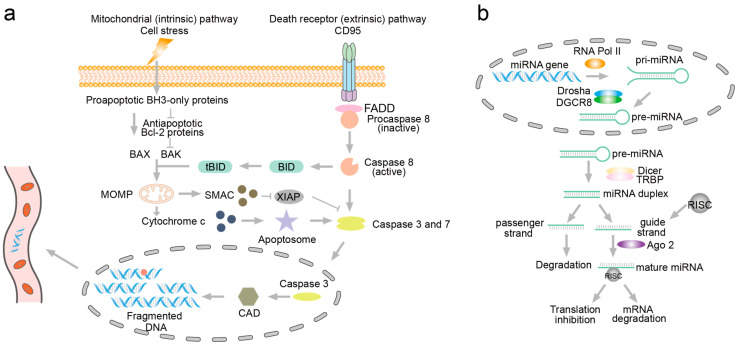
The fragmentation mechanism of DNA during apoptosis and the generation of miRNA. (**a**) Fragmentation of DNA. The activation pathways of caspases include the intrinsic (mitochondrial) pathway (left) and the extrinsic (death receptor) pathway (right). Upon activation within the nucleus, caspases further activate DNase (caspase-activated DNase, CAD). CAD then induces DNA fragmentation by converting DNA into mononucleosomal DNA, leading to a characteristic pattern of plasma DNA fragmentation that can be detected in the circulation. (**b**) The generation of miRNA. miRNA genes are transcribed into primary miRNAs (pri-miRNAs) in the nucleus, which are then processed by Drosha into precursor miRNAs (pre-miRNAs). The pre-miRNAs are exported to the cytoplasm and further processed by Dicer into mature, single-stranded miRNAs that regulate gene expression by targeting mRNAs.

Apoptosis, a programmed cell death process, involves specific degradation and fragmentation of nucleic acids mediated by intracellular enzymes, particularly nucleases (Figure 1a) [26]. External signals, such as apoptosis-inducing factors or cytokines, as well as internal signals like DNA damage or oxidative stress, initiate apoptosis pathways. These signals activate caspases, a family of cysteine proteases crucial in apoptosis, which, in turn, activate caspase-activated DNase (CAD). Normally bound to its inhibitor (iCAD), CAD becomes active upon cleavage of chromosomal DNA. CAD preferentially cleaves DNA at linker regions between nucleosomes, resulting in DNA fragments typically sized in multiples of 180–200 base pairs. Recent studies by Mao et al. further elucidate that cfDNA fragmentation correlates with apoptosis processes [27]. Using HL60 cells as a model, Mao et al. demonstrated that cfDNA fragmentation occurred both intracellularly and extracellularly during apoptosis. They observed a preference for C-terminal fragments in the supernatant and sediment of apoptotic HL60 cells. This phenomenon not only aids in early cancer detection but also facilitates monitoring cancer progression. The detection and analysis of ctDNA fragments in liquid biopsy offer insights into tumor dynamics, treatment response, and the emergence of resistance mutations, thereby guiding personalized treatment strategies in oncology.

MicroRNAs (miRNAs) have garnered significant attention as potential biomarkers in cancer liquid biopsy due to their stability, detectability, and specific expression patterns in various cancer types [28,29,30]. miRNAs are endogenous, single-stranded, non-coding RNA molecules typically 21–22 nucleotides long, processed from hairpin structure precursors [31]. In the nucleus, miRNA genes are transcribed into long primary miRNAs (pri-miRNAs) [32]. The Drosha/DGCR8 complex then processes pri-miRNAs into precursor miRNAs (pre-miRNAs) of approximately 60–70 nucleotides in length. Subsequently, pre-miRNAs are transported from the nucleus to the cytoplasm with the assistance of Exportin-5. In the cytoplasm, Dicer further cleaves pre-miRNAs into mature miRNAs, which are then incorporated into the RNA-induced silencing complex (RISC). Mature miRNAs within RISC regulate gene expression by binding to target mRNAs (Figure 1b) [33]. They play crucial regulatory roles in gene expression and are involved in diverse cellular processes, including proliferation, differentiation, and apoptosis. In cancer, dysregulation of miRNAs is a common phenomenon. Aberrant expression of miRNAs can act as oncogenes or tumor suppressors, contributing to carcinogenesis by modulating critical pathways involved in cell cycle control, apoptosis, DNA repair, and metastasis. The unique expression profiles of miRNAs across different cancer types and stages make them attractive candidates for biomarker development in liquid biopsy assays. miRNAs are remarkably stable in biofluids such as blood, urine, saliva, and cerebrospinal fluid due to their association with protein complexes or encapsulation in exosomes or microvesicles [34]. This stability enables their detection and quantification even after prolonged storage, making them suitable for non-invasive liquid biopsy [35,36].

### 2.2. Physical Degradation

Physical degradation poses a significant challenge in nucleic acid detection, arising from routine experimental manipulations such as pipetting, solution dilution, vortexing, agitation, freeze–thaw cycles, sonication, and filtration [37]. These processes exert mechanical and shear forces on nucleic acid molecules, resulting in chain breakage and fragmentation [38]. Specifically, during aspiration and dilution, the flow and mixing of liquids impose shear forces on nucleic acid molecules. Vortexing and agitation enhance molecular motion and collisions through mechanical vibration, increasing the risk of nucleic acid strand breakage. Freeze–thaw cycles subject nucleic acids to physical squeezing from ice crystal formation and melting. Sonication and filtration exert direct pressure or sound waves that can destabilize nucleic acid structures. Cumulatively, these physical forces can fragment intact nucleic acid molecules into smaller pieces or even compromise their original structure and function. To delve deeper into the effects of physical degradation on nucleic acid molecules, Yoo et al. conducted a series of experiments using linearized λ-phage virus DNA as a model [39]. They employed sensitive flow cytometry analysis to observe and quantify the extent and characteristics of DNA fragmentation under typical experimental conditions. These experiments not only confirmed the damaging effects of physical operations on DNA molecules but also detailed the varying degrees of fragmentation caused by different procedures. This research provides crucial insights for assessing the potential risks of nucleic acid degradation in experimental settings and serves as a basis for optimizing nucleic acid detection methods and procedural protocols. Specifically, during aspiration and dilution, the flow and mixing of liquids impose shear forces on nucleic acid molecules. Vortexing and agitation enhance molecular motion and collisions through mechanical vibration, increasing the risk of nucleic acid strand breakage. Freeze–thaw cycles subject nucleic acids to physical squeezing from ice crystal formation and melting. Sonication and filtration exert direct pressure or sound waves that can destabilize nucleic acid structures. Cumulatively, these physical forces can fragment intact nucleic acid molecules into smaller pieces or even compromise their original structure and function.

### 2.3. Heat-Induced Degradation

Heat-induced degradation poses significant challenges to nucleic acid detection methodologies, impacting both DNA and RNA molecules in various experimental and practical contexts. DNA molecules are highly susceptible to degradation under elevated temperatures, particularly above 100 °C. This thermal stress induces structural changes such as denaturation, depurination, depyrimidination, and loss of secondary structures [40]. Such alterations not only compromise the integrity of the DNA molecule but also hinder accurate detection and analysis. Kibayashi et al. conducted experiments involving heat-treated DNA samples extracted from oral swabs and cell lines, where varying heat exposure times were used to achieve different degrees of DNA fragmentation [41]. This approach is crucial for studies like Amplified Fragment Length Polymorphism (AFLP) analysis, where degraded DNA samples are preferred for assessing genetic diversity and variation. RNA molecules exhibit even greater sensitivity to temperature fluctuations compared to DNA [42]. Yamanaka et al. demonstrated this sensitivity through experiments focusing on zebrafish environmental DNA (eDNA) and environmental RNA (eRNA) in recirculating water tanks [43]. Their findings highlighted that higher water temperatures accelerate the degradation of both eDNA and eRNA, with eRNA degrading faster than eDNA under similar conditions. Hence, understanding the kinetics and extent of DNA and RNA degradation due to heat is essential for developing robust detection methods that can withstand environmental and processing conditions.

## 3. Detection Methods for Fragmented Nucleic Acid Molecules

The detection of fragmented nucleic acid molecules is crucial for advancing our understanding of various biological processes and disease states [44]. Recent technological advancements have significantly improved the sensitivity and precision of these detections. Among the most impactful methods are next-generation sequencing (NGS), high-resolution melting (HRM) analysis, molecular probes, and CRISPR-based approaches. NGS offers a high-throughput capability to sequence millions of DNA or RNA fragments simultaneously, providing detailed insights into fragmented nucleic acid profiles. The high resolution of HRM makes it suitable for applications such as SNP analysis and the assessment of nucleic acid integrity.

In addition to NGS and HRM, molecular probes and CRISPR-based technologies have emerged as powerful tools for detecting fragmented nucleic acids. Molecular probes, including linear and locked nucleic acid probes, can specifically bind to target sequences, enabling precise identification and quantification of fragmented molecules [45]. These probes are often used in combination with ligation or amplification reactions to provide high-sensitivity measurements in complex samples. Meanwhile, CRISPR-based detection methods utilize the targeted cleavage activity of Cas proteins (such as Cas9) and the collateral cleavage activity of Cas12 and Cas13 to generate detectable signals in the presence of target fragments [46,47]. These innovative approaches offer exceptional sensitivity and specificity, making them suitable for diagnostic applications and environmental monitoring.

### 3.1. Next-Generation Sequencing Technology

**Figure 2:**
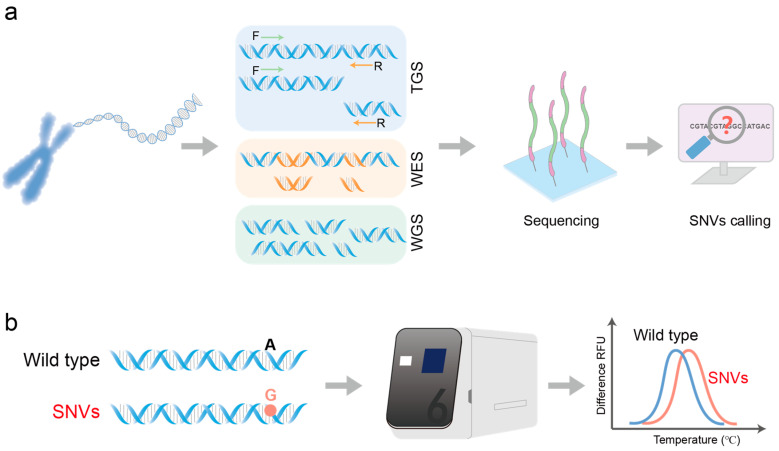
Schematic presentation of detecting SNV in nucleic acid molecules using next-generation sequencing (NGS) technology and high-resolution melting curves. (**a**) Targeted genome sequencing (TGS), whole exome sequencing (WES), and whole genome sequencing (WGS) are used to detect SNVs in nucleic acid molecules. TGS is captured by PCR amplification or DNA enrichment methods to obtain the genomic sequence of interest. WES focuses on the protein-coding regions of the genome. WGS sequences all DNA in the genome of an organism. (**b**) Detecting SNV through high-resolution melting curve analysis. Fluorescence quantitative PCR Instrument is used to detect dsDNA with different base compositions, and SNV is distinguished by the unique Tm curve of DNA samples.

NGS technology represents a pivotal method for detecting SNVs in nucleic acid molecules, capable of identifying unknown single nucleotide variations, and it offers significant advantages in sequencing speed and throughput. Current NGS platforms, such as Illumina and DNBSEQ, excel with paired-end sequencing lengths typically not exceeding 300 bp, ideally suited for fragmented nucleic acid molecule detection. This technology is widely applied in various contexts, including targeted genome sequencing (TGS), whole exome sequencing (WES), and whole genome sequencing (WGS) (Figure 2a).

TGS typically focuses on specific genomic regions, such as well-known driver genes or clinically actionable genes, using high-throughput sequencing to identify sequencing variations, and has been widely applied in cancer research and clinical trials [48]. For instance, it has been utilized to detect KRAS mutations in ctDNA from pancreatic cancer patients, highlighting its utility in molecular profiling and treatment selection [49]. These targeted regions are often amplified via PCR or captured through DNA enrichment methods to obtain the genomic sequences of interest. Despite its cost-effectiveness, PCR introduces several challenges in variant detection. Firstly, PCR can induce mutations during the amplification process [50], potentially impacting the accuracy of SNV detection. Secondly, the sequence bias of PCR amplification [51,52] and different break positions and break lengths of fragmented ctDNA molecules [53] may lead to different copy numbers or mutation frequencies. In the context of SNV detection, TGS offers advantages by concentrating sequencing efforts on predefined genomic regions, thereby enhancing sensitivity and specificity compared to whole-genome approaches. However, addressing PCR-induced artifacts and accounting for the complexities introduced by fragmented nucleic acids are critical for reliable variant calling.

WES is a targeted sequencing method that focuses on the protein-coding regions of the genome, circumventing issues associated with PCR-based approaches for acquiring specific nucleic acid sequences. However, a significant challenge of WES is that its sequencing coverage in the target exonic region is uneven, resulting in batch effects, which can introduce differences between different laboratories, operators, and sequencing batches [54]. In contrast, WGS offers a more uniform coverage across the entire genome and is more effective in detecting potential pathogenic mutations within the regions targeted by WES despite requiring more sequencing resources. Belkadi et al. discovered that within identical samples, about 3% of coding variants (650 SNVs) were detected by WGS and missed by WES [55].

Regardless of the sequencing approach chosen, NGS methods must confront inherent sequencing error rates of no more than 1% [56]. Overcoming these challenges necessitates the implementation of robust bioinformatics pipelines and rigorous validation strategies to ensure the fidelity and reliability of identified variants. Xiao et al. utilized paired tumor–normal cell lines to analyze the impact of various biological sample types, library preparation methods, sequencing platforms, and bioinformatics analysis pipelines on mutation detection and provided recommended practices for utilizing NGS to achieve reproducible and accurate cancer mutation detection [57].

### 3.2. High-Resolution Melting Curves

Due to the disruption of hydrogen bonds between complementary bases at high temperatures, double-stranded DNA (dsDNA) is denatured into two single-stranded DNA (ssDNA) molecules [58]. The temperature at which half of the dsDNA transitions from a double helix to a random coil of single strands is defined as the melting temperature (Tm). Tm is influenced by the specific sequence of DNA, causing dsDNAwith different base compositions to exhibit distinct Tm values. Consequently, by analyzing characteristic melting curves of DNA samples, SNVs can be precisely identified and differentiated based on their unique Tm profiles (Figure 2b) [59]. High-resolution melting (HRM) is a technique based on liquid-phase melting analysis that integrates PCR technology to precisely monitor the fluorescence of DNA-binding dyes as dsDNA segments melt at high temperatures, enabling the detection of SNVs within dsDNA [60]. Reed et al. investigated the capability of HRM to identify heterozygous single-base changes across PCR products ranging from 50 to 1000 bp [61]. Their findings reveal that HRM achieves high accuracy in identifying variants shorter than 300 bp, correctly identifying all heterozygotes. However, for heterozygotes with mutations near the center ranging from 400 to 1000 bp, sensitivity and specificity were 96.1% and 99.4%, respectively. Myrick et al. integrated extreme real-time PCR and high-speed melting analysis to achieve the amplification and identification of infectious disease sequences and genotyping of human SNVs from genomic DNA, all within a mere 52 to 87 s [62]. The length of DNA sequences ranges from 57 to 83 bp. However, this testing method relies on expensive equipment and still faces challenges with PCR when dealing with samples that have inconsistent break positions.

### 3.3. Hybridization Probe Technology

Hybridization probes represent a cornerstone in the field of nucleic acid detection, offering a robust method to identify specific sequences within complex genetic material [63,64]. They are designed to capitalize on the principle of complementary base pairing, enabling precise recognition and localization of target nucleic acids through controlled hybridization events. Two primary categories of hybridization probes are commonly employed: linear probes and padlock probes [65,66].

**Figure 3:**
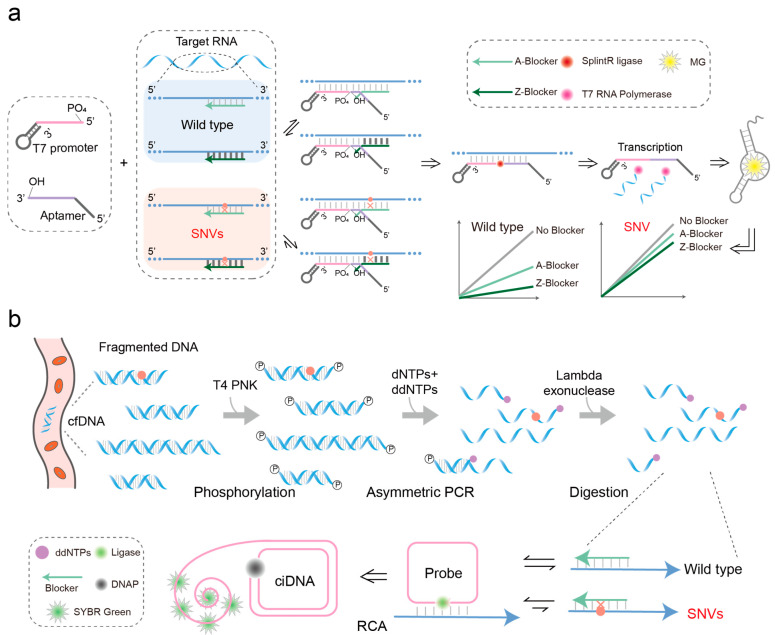
Hybridization probe technology is used to detect SNV in nucleic acid molecules. (**a**) Linear probe detection technology. This process involves two linear probes and a toehold blocker. Firstly, both linear probes bind to the target sequence and effectively replace the complementary binding blocker through the toehold mechanism. Secondly, the two linear probes are ligated by a ligase. Finally, the RNA aptamer encoded on the linear probe is transcribed and emits a fluorescent signal upon binding to malachite green. The Z base replaces the A base in the blocker, which can pair with the T base to increase the binding affinity of the blocker to the target sequence and improve the specificity of binding. (**b**) Padlock probe technology. Combining the toehold-mediated strand displacement reaction with a locking probe to detect SNVs in the human genome. Firstly, introduce ddNTP into the PCR system to enrich cfDNA as comprehensively as possible. Then, the blocker binds to the DNA template chain, and finally, the padlock probe preferentially binds to the template through its toehold region, gradually replacing the blocker and then replacing it to bind to the template. Under the action of T4 DNA ligase, circular single-stranded DNA is generated.

Linear probes are typically short, single-stranded oligonucleotides engineered to bind specifically to complementary sequences within the target nucleic acid. This straightforward design facilitates the detection of specific genetic markers or sequences of interest by virtue of their sequence complementarity. In the context of SNV detection, shorter linear probes exhibit higher sensitivity due to the reduced stability of probe–target binding upon a single base mismatch [67]. However, the short length of these probes also increases the likelihood of non-specific binding to non-target sequences, leading to false positive results and compromising the accuracy of SNV analysis. On the other hand, the employment of dual-linearity probes for detection offers distinct advantages, as signal generation is contingent upon the simultaneous binding of both strands of the probe to the target DNA. This design feature enhances the specificity of SNV detection, thereby improving the overall reliability of genotyping assays. Gao et al. achieved SARS-CoV-2 genotyping at ~10 pM through the use of entropy-driven footpoint blockers to control the assembly of dual probes on the target, combined with ligation and transcription reactions (Figure 3a) [68]. In this method, linear probes replace blockers bound to the target via a toehold-mediated strand displacement reaction, thereby enabling the ligation between two probes to initiate transcription reactions that emit fluorescent signals. The thermodynamic stability of blocker binding to both the target and mutated target sequences is crucial for determining detection sensitivity. To bolster SNV detection sensitivity, Kang et al. innovatively substituted adenine (A) bases in the blocker with 2,6-diaminopurine (Z) bases, which can form triple hydrogen bonds with thymine (T) bases (Figure 3a) [69]. This modification increases the affinity of blockers for target sequences, which is particularly beneficial in contexts where high specificity is required. The enhanced stability conferred by Z bases improves the precision of detection, minimizing false positives and ensuring reliable identification of SNVs.

A padlock probe is a single-stranded linear DNA (28–188 nt) modified with a phosphate group at the 5′ end [70]. It consists of two main parts: complementary segments at the 5′ and 3′ ends, each approximately 15–20 nt long, which hybridize with the target sequence to form a contiguous structure. The middle region is an unrelated DNA sequence that can be used for primer design in downstream rolling circle amplification (RCA) reactions or incorporate functional nucleic acid sequences such as adapters, G-quadruplex motifs, and DNAzyme sequences. Padlock probes are designed to specifically bind to target sequences, facilitating the formation of circularized DNA molecules upon hybridization. Traditional padlock probes used for SNV detection primarily rely on DNA ligase fidelity. However, it has been reported that DNA ligase can still catalyze the formation of phosphodiester bonds between the 5′ phosphate and 3′ hydroxyl groups of padlock probes, even in the presence of mismatches, leading to potential false-positive or false-negative results. Researchers have explored various strategies to enhance the specificity of detection, such as redesigning padlock probes or substituting ATP with ATP-αS to improve the substrate specificity of T4 DNA ligase [71,72,73]. However, these approaches often compromise ligation efficiency, resulting in reduced overall reaction yield and affecting detection sensitivity. Gao et al. combined the high specificity of toehold-mediated strand displacement probes with the signal amplification capability of padlock probes, toehold-controlled ligation, and transcription (TLT) to detect SNVs relevant to cancer occurrence, development, treatment, and prognosis in the human genome (Figure 3b) [74]. When facing the challenge of detecting fragmented cfDNA segments with variable lengths, which can impact the accuracy of mutation frequency when using PCR for DNA amplification, Gao et al. introduced ddNTPs into an unbalanced PCR system to enrich cfDNA as comprehensively as possible [74]. By integrating this approach with the toehold-assisted padlock probe method, they achieved precise detection of highly fragmented cfDNA.

### 3.4. CRISPR/Cas System

The complexes of programmable clustered regularly interspaced short-palindromic repeats (CRISPR)-associated nucleases (Cas) and their corresponding nucleic acids demonstrate high efficiency and specificity toward DNA/RNA targets, highlighting the substantial potential in nucleic acid detection [46]. CRISPR-Cas systems are categorized into two major classes based on the composition and number of effector proteins involved in biochemical reactions: Class 1 system requires the cooperative action of two or more effector proteins to function, while Class 2 systems can operate effectively with a single effector protein. Class 2 system, exemplified by Cas9 with its targeted cleavage ability, and Cas12 and Cas13 with additional collateral cleavage activities, are highly favored in nucleic acid detection due to their straightforward operation and rapid response [75,76,77]. 

**Figure 4:**
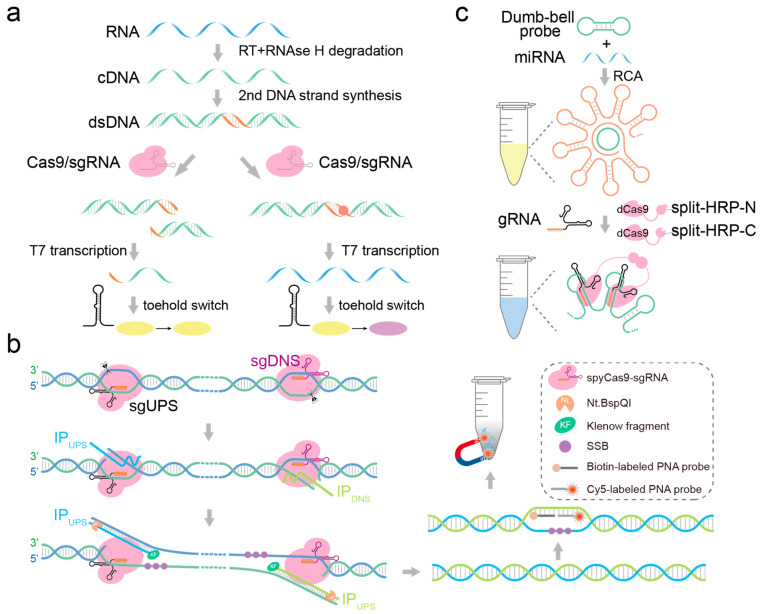
Detecting fragmented nucleic acid molecules based on the CRISPR-Cas9 system. (**a**) CRISPR-Cas9 system: targets and cleaves the target dsDNA. (**b**) CRISPR-Cas9n system: targets the dsDNA and cleaves its complementary or non-complementary strand. (**c**) CRISPR-dCas9 system: targets the target sequence without a cleavage reaction.

In vitro, CRISPR-Cas9 forms a complex guided by crRNA-trancrRNA (gRNA) that specifically targets double-stranded DNA, causing double-strand breaks, making it widely applicable in nucleic acid detection. CRISPR-Cas9 does not cleave target sequences when the PAM region experiences single-base mismatches. Building on this concept, Pardee et al. pioneered the use of Cas9 protein in nucleic acid biosensing, developing a Zika Virus variant genotyping method known as NASBACC with single-base resolution [78]. This method initially employs NASBA technology for isothermal amplification of viral RNA molecules, followed by specific recognition and cleavage of target sequences by CRISPR-Cas9, thereby preventing activation of the toehold switch. In scenarios where the target lacks a PAM sequence, RNA products activate the toehold switch, triggering a colorimetric response (Figure 4a). However, this method requires the enrichment of nucleic acid in the early stage, which is complicated, and it is easy to introduce mutations in the enrichment process. The formation of DNA R-loops and double-strand breaks in dsDNA by Cas9 is mediated through the separate cleavage of the non-target and target strands by its RuvC and HNH domains, respectively [79,80]. Programmable Cas9 nickases (Cas9n), capable of generating strand-specific cuts in dsDNA, are achieved through the introduction of D10A mutation in the RuvC domain or the H840A mutation in the HNH domain [81,82]. Zhou et al. developed a CRISPR-Cas9n-triggered endonuclease-mediated strand displacement amplification (CRISDA), a typical Cas9n detection scheme [83]. In CRISDA reactions, Cas9n first generates a cleavage at the dsDNA target site, followed by a linear strand displacement reaction and exponential amplification triggered by a pair of initiating primers (IP). Each IP primer consists of a single Nb.BbvCI endonuclease cleavage site, a central hybridization region, and a 3′ overhang complementary to the displaced non-target strand. Experimental results demonstrate that IP primers efficiently initiate exponential amplification as long as the melting temperature of the 3′ overhang exceeds 50 °C without requiring further optimization (Figure 4b); the experimental data also proved that Cas9-mediated CRISDA has relatively high sensitivity and specificity in DNA detection, and can detect single-base mutations; however, like PCR-based amplification, CRISDA also has an amplification bias when performing amplification. If mutations occur simultaneously at the RuvC D10A and HNH H840A sites of Cas9, it results in the formation of a nuclease-deficient Cas9 (dCas9) [84]. The dCas9-gRNA complex retains the capability to bind to the target dsDNA but loses its ability to cleave DNA. Qiu et al. developed a detection platform for microRNAs (miRNAs) with lengths ranging from 18 to 24 nucleotides using the dimerization principle, termed RCA-CRISPR-split-HRP (RCH) [85]. The RCH method initiates with the specific binding of target miRNA to probes, triggering strand displacement reactions that form structures conducive to rolling circle amplification (RCA). Subsequently, guided by specific sgRNA, split-HRP-dCas9 fusion proteins are recruited to the vicinity of RCA products, activating HRP enzymatic activity, thereby generating a colorimetric response (Figure 4c). 

**Figure 5:**
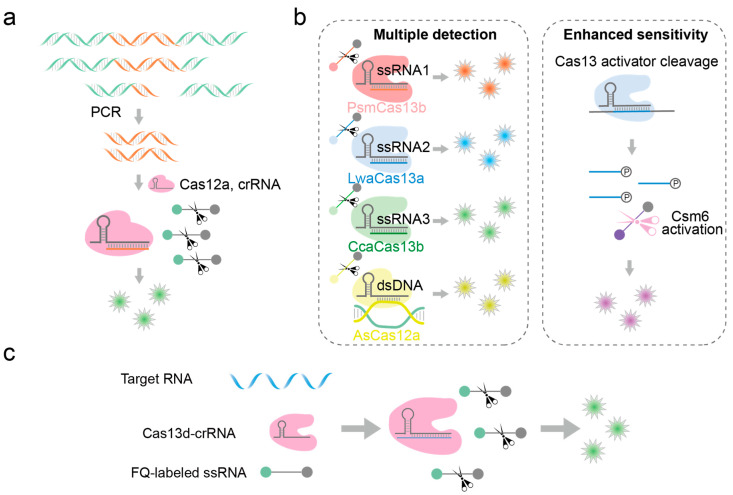
Detection of fragmented nucleic acid molecules based on CRISPR-Cas12 and CRISPR-Cas13 systems. (**a**) HOLMES: Firstly, PCR reaction is used to enrich the nucleic acid to be detected. Then, the CRISPR-Cas12a system targets and binds to the nucleic acid to be detected and finally triggers a trans-cleavage reaction to cleave the fluorescent probe and release a fluorescent signal. (**b**) SHERLOCKv2: LwaCas13a, PsmCas13b, CcaCas13b, and AsCas12a simultaneously detect multiple viruses, among which Csm6 can cleave reporter molecules after being activated by cleavage byproducts. (**c**) Nucleic acid detection technology based on CRISPR-EsCas13d and CRISPR-RspCas13d.

Cas12 and Cas13 are Cas effectors guided by RNA to specifically recognize and cleave dsDNA and RNA, respectively, and they exhibit collateral cleavage activity toward ssDNA and RNA, respectively [86]. Their collateral cleavage activity can be used as signal amplifiers in nucleic acid detection to improve the sensitivity of the analysis [87]. HOLMES, a nucleic acid detection platform based on Cas12a, was proposed by Li et al. in 2018 (Figure 5a) [88]. It utilizes truncated crRNA (16 and 17 nt) to recognize target sequences, imparting exceptionally high cleavage specificity. This platform can detect SNPs even when the target site is distant from the PAM site. However, it relies on PCR amplification to introduce the PAM sequence into the target, which also faces challenges associated with PCR itself. Utilizing the non-specific RNA cleavage capability of Cas13a upon target binding, Zhang et al. developed a nucleic acid detection technology based on CRISPR-Cas13a in 2017, named “SHERLOCK” [76]. Initially, a short single-stranded RNA probe was designed with a fluorophore at one end and a quencher at the other. Upon the formation of the Cas13-crRNA-RNA complex in the presence of target RNA, the Cas13 protein was activated to catalyze the trans-cleavage of the RNA probe, leading to fluorescence emission indicative of the presence of the target nucleic acid probe. This technology enabled rapid and sensitive detection of Zika virus and Dengue virus, achieving sensitivity levels down to aM concentrations and demonstrating the capability to identify single-base mutations, and this technology still needs to enrich nucleic acids in the early stage. In 2018, Zhang et al. further refined the “SHERLOCK” technology, leveraging Cas13a, Cas13b, Cas12a, and Csm6 proteins with varying cleavage specificities and nucleotide preferences, thereby enhancing the capacity of the platform for simultaneous detection of multiple viruses, as indicated in Figure 5b [89]. Regarding the CRISPR-Cas system, Cas13d, compared to Cas13a, is approximately 20% smaller in terms of amino acid content. Exploratory research has revealed that EsCas13d and RspCas13d, unlike other variants, do not rely on Protospacer Flanking Sequences (PFS) for the targeting of the respective guide RNA (crRNA) to the target RNA. This find imparts greater flexibility in the design of crRNA sequences, enabling them to be more adaptable to various target RNA molecules [90]. Qiao et al. were the first to use an EsCas13d and RspCas13d-based nucleic acid detection method for SNV identification [91]. When the Cas13d-crRNA complex specifically binds to the target ssRNA, the trans-cleavage activity of Cas13d is activated, leading to the cleavage of a short ssRNA reporter gene and the generation of a fluorescence signal (Figure 5c). This method enables the sensitive detection of SNVs with allele frequencies as low as 0.1%. 

In order to assist the introduction and discussion, we summarize the detection substrates and their advantages and disadvantages of various detection techniques, as shown in Table 1.

## 4. Conclusions and Outlooks

With the rapid advancement of science and technology, key technologies, such as Next-Generation Sequencing (NGS), High-Resolution Melting (HRM) analysis, molecular probes, and CRISPR-based methods, have revolutionized the field of fragmented nucleic acid detection. NGS, with its high-throughput advantage, can simultaneously process millions of DNA or RNA fragments, providing us with detailed and in-depth insights into fragmented nucleic acid profiles. However, despite its outstanding capabilities, NGS still faces challenges such as high error rates and considerable costs. Additionally, NGS and many other traditional techniques rely on PCR for target amplification, which not only increases operational complexity but can also affect the accuracy and reliability of amplification. HRM analysis relies on expensive machinery. As technology progresses, we must continually optimize and improve these tools to meet the growing demands. Molecular probes play a crucial role in the identification and quantification of fragmented molecules due to their high specificity and precision. These probes, designed with great care, can precisely bind to target sequences, providing reliable data support. CRISPR-based methods, leveraging the targeted cleavage activity of Cas proteins, generate detectable signals, exhibiting extremely high sensitivity and specificity. However, all these methods still face a series of challenges on the road to broader application, including enzyme specificity, cost-effectiveness, and operational simplicity. Fluorescent groove binders or intercalators with sequence selectivity hold promise for single nucleotide variant detection due to their high sensitivity and ease of use [92]. However, the effectiveness of these probes may be limited to specific sequences, reducing their applicability across different targets.

Looking ahead, in the field of single-nucleotide variation (SNV) detection, it needs to aim to avoid using PCR for target amplification to reduce operational complexity and improve accuracy. Additionally, to meet the needs of point-of-care testing (POCT), simple, rapid, and low-cost detection methods need to be developed. These new methods will integrate the latest biotechnology, nanotechnology, and digital technology to achieve efficient and precise detection of SNVs. We believe that with continuous technological advancement and innovation, future SNV detection will become more convenient, economical, and efficient, providing stronger support for clinical diagnosis, disease treatment, and personalized medicine.

## Figures and Tables

**Table 1:** Detection substances and the advantages and disadvantages of various SNV detection methods.

Method	Technology	Detection Substances	Advantages	Disadvantages	Ref.
Next-generation sequencing technology	TGS	Specific genomic fragments	No prior knowledge of the position or identity of the variants	Inherent limitations of PCR and high sequencing error rate	[49]
	WES	Protein-coding regions of the genome		Batch effects and high sequencing error rate	[54]
	WGS	Whole genome		More sequencing resources and high sequencing error rate	[55]
High-resolution melting curves	HRM	DNA	Fast	Strong technical dependence and inherent limitations of PCR	[61,62]
Hybridization probe technology	TLT	DNA or RNA	Rapid, portable, room temperature, device-independent	Requires prior knowledge of wild DNA sequences	[68]
	Z/TLT				[69]
	Toehold-assisted padlock probe systems				[74]
CRISPR/Cas system	NASBACC	DNA	Rapid, portable, high sensitivity, and specificity	Requires prior knowledge of wild DNA sequences and inherent limitations of PCR	[78]
	CRISDA	DNA			[83]
	RCH	DNA			[85]
	HOLMES	DNA			[88]
	SHERLOCK	RNA			[89]
	EsCas13d and RspCas13d-based detection method	RNA			[91]

## Data Availability

The raw data supporting the conclusions of this article will be made available by the authors on request.
